# Supplementary material for: Vulnerability of power distribution networks to local temperature changes induced by global climate change
Source: Nat Commun. 2025 Jun 2;16:5116. doi: 10.1038/s41467-025-59749-4 (PMC12130186; doi:10.1038/s41467-025-59749-4)
Supplement: Supplementary file 1 — Supplementary Information [file 41467_2025_59749_MOESM1_ESM.pdf]

Supplementary Information for

Vulnerability of Power Distribution Networks to Local Temperature Changes Induced by Global Climate Change

Kishan Prudhvi Guddant†, Lin Chen†, Yang Weng*, Yang Yu*

† The authors contributed equally

*Corresponding author. Email: yang.weng@asu.edu and yangyu@cup.edu.cn

This PDF file includes:

Supplementary Notes 1-7
Supplementary Methods 1-2

Supplementary Reference

Supplementary Note

Supplementary Note 1. Overview of Supplementary Information

In Supplementary Information (SI), we will cover a range of basic topics, from the definition of distribution grids, to the basic concepts of per unit ($p.u.$). To facilitate the analysis and control of distribution grids, a common method of measurement is the per unit ($p.u.$) system, which is based on a normalized scale that simplifies the calculations involved in power flow and fault analysis. In addition to the $p.u.$ system, there are different types of distribution networks, including mesh and tree networks, which vary in their structure and complexity.

In SI, we will explore several key aspects of distribution grids, including the impact of temperature on the safe operation boundary, the uncertainty of risk analysis, and the climate change models that are used to simulate the behavior of distribution grids. Additionally, we will examine the power demand variations of distribution grids under different temperature conditions and provide data related to the distribution grid and load consumption. By examining these concepts, we aim to provide a comprehensive understanding of the key factors of the impact of climate change on distribution networks.

We examined the distribution grid (DG)'s blackout risk based on the two scenarios from the years 1950 to 2100 according to the local temperature data from the U.S. Geological Survey (USGS) National Climate Change Viewer (NCCV) [1]. In the USGS NCCV database, the temperature before 2005 was obtained from the historical records while the temperature afterward is projections according to the 20 models based on Coupled Model Intercomparison Project Phase 5 (CMIP5). According to the report from the National Conference of State Legislation (NCSL), 60% of the DG lines were constructed more than a half-century ago [2]. Therefore, we examined the DG system's vulnerability to change since 1950. We particularly focused on the risk change by 2050 while the DG system usually serves for decades. We also projected the blackout risk after 2050 until 2100 in order to understand the climate change's pure effect in the long term and clarify when the risk will non-linearly grow. The climate change process is expected to accelerate in the second half of this century. It is necessary to predict the impact of the rapid climate change progress. In particular, whether the climate change's impact will diverge by climate scenarios is critical. Thus, most studies about climate change and its impact predict the situations until 2100 [3, 4]. In our research, predicting the blackout risk after 2050 can further reveal the vulnerability of the current real-world DG system during the rapid climate change process.

Supplementary Note 2. Introduction of distribution grid

In a real power grid, the transmission grid (TG) injects power into the substation via transmission lines. From the substation to the customer terminal, all belong to the distribution grid (DG). The substation converts the high-voltage power into medium-voltage power, which is transmitted to the nodal transformers via medium-voltage distribution lines. The transformer is converted into low-voltage power and transmitted to the end user via low-voltage distribution lines. In this paper, we aggregated the demands beneath a transformer into a single equivalent load. Therefore, the node of DG in the paper denotes a substation and some transformers connected to equivalent loads. The DG architecture is shown in Supplementary Fig. 1. The schematic only shows the radial structure, but there exists the mesh-type structure, which operates on the same principles.

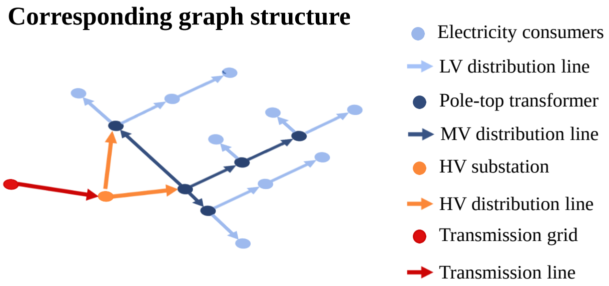

Supplementary Fig. 1 Electricity Distribution Network Overview. The image shows the flow of electricity from generation to consumers. Electricity Consumers are end-users like homes and businesses. Low voltage (LV) distribution lines deliver low-voltage power to them. Pole-top Transformers convert medium voltage (MV) to LV. MV Distribution Lines carry electricity from substations to transformers. High voltage (HV) substations step down for local distribution. HV Distribution Lines link substations to the Transmission Grid, which transports power over long distances via Transmission Lines. This network ensures efficient electricity delivery.

Supplementary Note 3. Definition of per unit ($p.u.$)

In the power systems analysis field of electrical engineering, per unit is the expression of system quantities as fractions of a defined base value quantity [5]. Different types of quantities are labeled with the same symbol ($p.u.$). Therefore, it

is possible to represent distribution networks of different voltage levels with the same analysis. A $p.u.$ value can readily be reverted back to a voltage, power, or any other value by multiplying by the base value (the voltage of the distribution network).

Supplementary Note 4. Toy example of mesh and tree networks

Specifically, the 3-bus mesh and tree networks presented in Supplementary Fig. 2a and Supplementary Fig. 2d are used as examples whose transmission lines are purely resistive i.e., $\overline{Y}_{12} = \overline{Y}_{23} = \overline{Y}_{13} = 1p.u.$. For both mesh and tree networks, Node 1 has the generator, and Nodes 2 and 3 have the users whose power demands are indicated by P_2, and P_3, respectively.

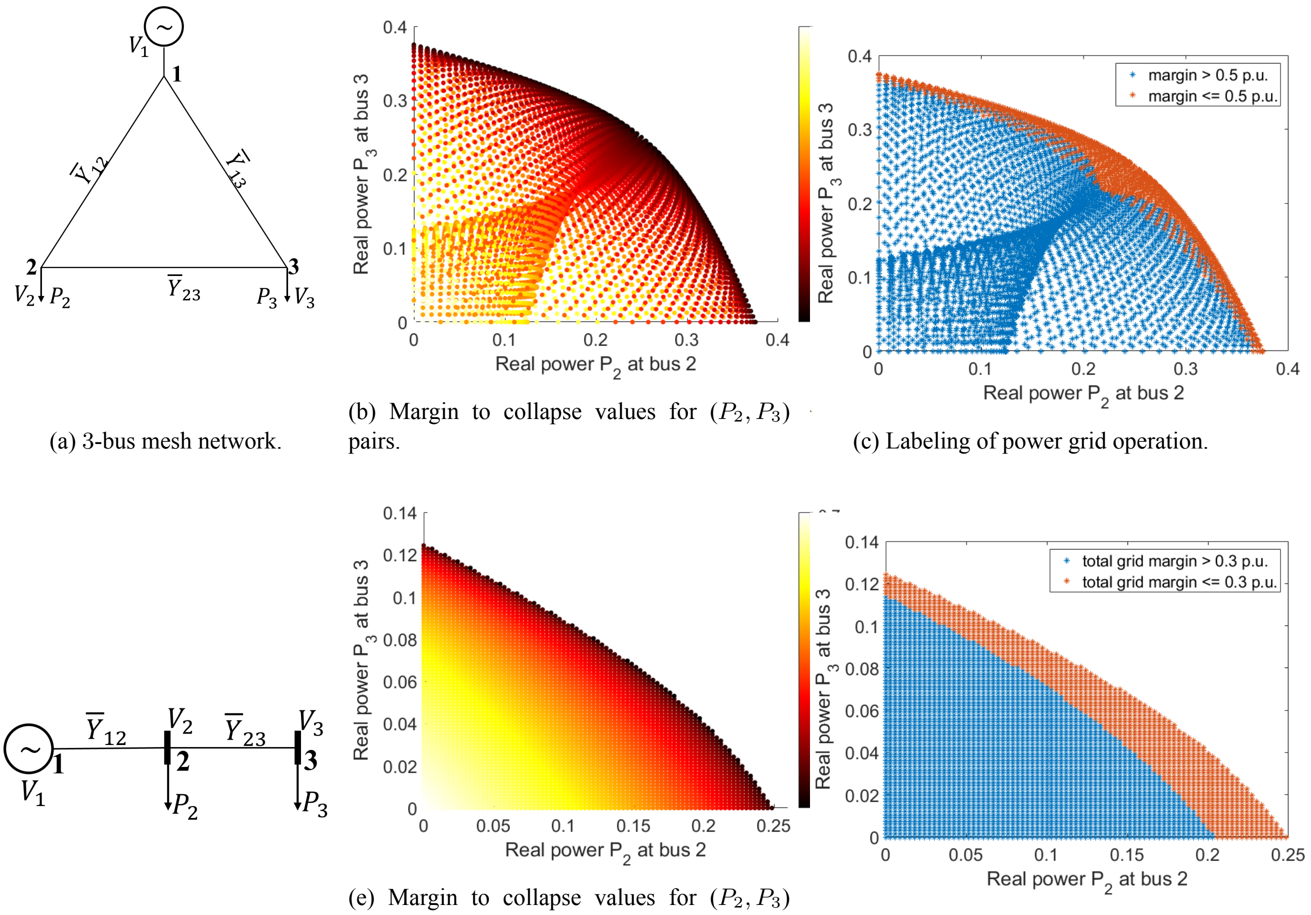

(a) 3-bus mesh network. (b) Margin to collapse values for (P_2, P_3) pairs. (c) Labeling of power grid operation.

(d) 3-bus tree network (e) Margin to collapse values for (P_2, P_3) pairs. (f) Labeling of power grid operation.

Supplementary Fig. 2 **Mesh and tree network example: distribution grid (DG)'s margin values for system collapse and labeling the desired region of DG operation. (a)** A 3-bus mesh type network and **(d)** A three bus tree type network with two consumers located at Nodes 2 and 3 whose demands are given by P_2 and P_3, respectively. **(b) and (e)** Margin to collapse value for every possible pair in (P_2, P_3) plane represented using the color bar. Note that the margin-to-collapse values of the data points on the border are zeros. The margin to collapse values of the data points (in (P_2, P_3) plane) decreases when moving towards the boundary. **(c) and (f)** The operation of DG is labeled as desired and undesired operating regions by using a threshold (α = 0.5 $p.u.$) on the margin to collapse values. For example, if the margin to collapse value of an operating condition (i.e., (P_2,P_3) pair value) is greater than 0.5 $p.u.$ (blue region) then it is desired and otherwise undesired (orange region). Note that when the threshold (α) is zero, the undesired region indicates the DG collapse events. Source data are provided as a Source Data file

The maximum power transfer limit (safe operation boundary) of a DG is dependent on its design and network structure. Using the Pareto front technique (Φ from Methods), the safe operation boundary can be charted for mesh and tree networks as shown in Supplementary Fig. 2b and Supplementary Fig. 2e, respectively. We observe in both Supplementary Fig. 2b and Supplementary Fig. 2e, a heat map is drawn for every possible pair of (P_2, P_3) and the color of each data point from color bar indicates the distance (margin) to system collapse. It can be noted that the pairs of (P_2, P_3) that contribute to system collapse (located on the outer boundary of these heat maps) in Supplementary Fig. 2b and Supplementary Fig. 2e have their margin to collapse (color bar) values (Φ values from Methods) to be zero. The operating points (P_2, P_3) beyond the boundaries in Supplementary Fig. 2b and Supplementary Fig. 2e result in a blackout as the power networks cannot host any additional real power demand beyond the safe operation boundary (SOB).

The various allocations of total line capacity are described in Supplementary Fig. 3. In this figure, there are ten different ways to allocate the total line capacity between the two lines in the tree DG. Each sub-figure shows the allocation of capacity on the two lines, with the corresponding risk level of the system shown in the title of the sub-figure. For example, in the fifth sub-figure, the total line capacity is evenly distributed between the two lines, resulting in a risk level of 43.76%. The lowest risk value is reached when the conductance of $\overline{Y}_{12}$ and $\overline{Y}_{23}$ are 3.4 and 2.6 respectively.

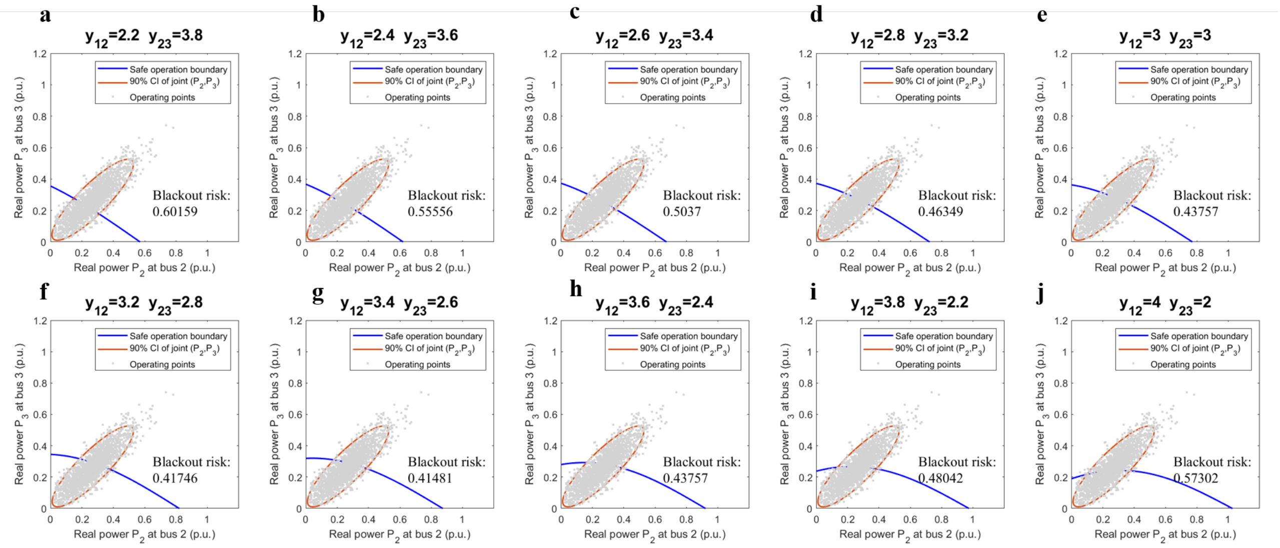

Supplementary Fig. 3 **The safe operating boundaries and corresponding blackout risks for different combinations of transmission line capacities in a 3-bus tree network with the same transmission capacity.** In each subfigure, y_{12} and y_{23} represent the conductance between lines 1 and 2, and the conductance between lines 2 and 3, respectively. Each panel shows results for different conductance combinations (y_{12} and y_{23}), with specific values labeled above each panel. The blue curve indicates the safe operation boundary (SOB) of the power grid. The scatter represents the operation points, and a 90% confidence ellipse is used to show the distribution characteristics of the data. It should be noted that the unit, per unit ($p.u.$), is the expression of system quantities as fractions of a defined base unit quantity. Source data are provided as a Source Data file.

Supplementary Note 5. Temperature effect on safe operation boundary

Every DG system has its maximal capacity of serving the demands of extracting power from the grid nodes. The maximal capacity of a DG system is contingent upon the capacities of transmission lines, transformers, and other facilities [6–8]. In a DG system that serves consumers who withdraw power from N nodes, the maximal capacity margin when the temperature is t can be captured by a function $\Phi[(P(t)_1, P(t)_2, \cdots, P(t)_n)]$ [9]. Here, $P(t)_i$ denotes the demand level on the Node i at temperature t [10]. When $\Phi[P(t)_1, P(t)_2, \cdots, P(t)_n] < 0$, the profile of nodal demands $(P_1, P_2, \cdots, P_n)$ can be safely served by the DG system. When $\Phi[P(t)_1, P(t)_2, \cdots, P(t)_n] > 0$, the grid will collapse while the blackout occurs. Thus, $\Phi[P|T_i, h] = 0$ represents the **safe operation boundary (SOB)**. In Supplementary Fig. 4a, we provide an example of the SOB of a triangle DG system serving the consumers located in two nodes. The area on the left-bottom of the SOB is the collection of all pairs of demands in Node 2 and 3 that can be safely served.

It is a random event whether the demands in a future hour h can lead to a blackout of the DG system. Nodal demand $P(T_i)_j$ is a random variable influenced by temperature and other factors. Each hour in the future, temperature T_i is uncertain. Given the same temperature, a consumer's electricity consumption still has randomness. Thus, the blackout probability of a DG system in hour h is defined in supplementary equation (1):

$$Risk_h = \sum_{i=1}^{t} p_h(T_i) \cdot p_h(\Phi[P(T_i)_1, P(T_i)_2, \cdots, P(T_i)_n)] \leq 0). \quad (1)$$

In Supplementary Fig. 4b, we show the samples of two groups of real-world consumers when the temperature is $30°C$. The green circle is the 90% quantile of the joint distribution of P_1 and P_2. If the two groups of consumers are respectively located on Nodes 2 and 3 of the triangle example DG system, the blue points are the demand profiles that can be safely served while the green points are the demand profiles that will trigger a blackout. Thus, according to the joint distribution of nodal demands, we are able to calculate the blackout probability of the DG system.

Climate change is going to reshape a DG system's probability of blackout by changing the level and pattern of consumer electricity demand. Due to climate change, extreme weather events such as heat waves and cold snaps will become more

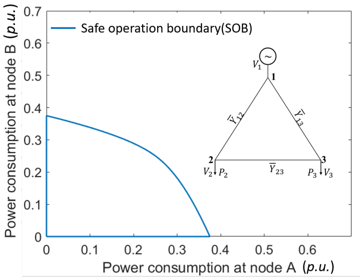

(a) Safe operation boundary (SOB) of the triangle shape 3 bus system.

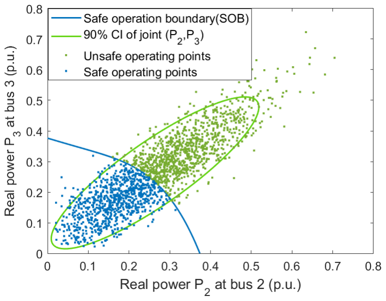

(b) Safe operation boundary (SOB) of the distribution grid and consumers' power demand.

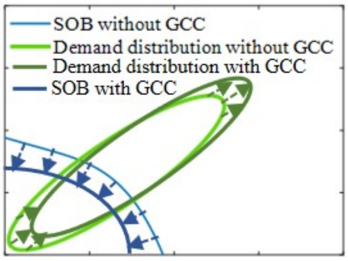

(c) Safe operation boundary (SOB), consumers' power demand before and after the influence of global climate change (climate change).

Supplementary Fig. 4 Illustration to calculate the probability of distribution grid (DG) blackout due to global climate change. The considered DG is a triangle shape three bus system whose distribution lines are purely resistive i.e., $\overline{Y}_{12} = \overline{Y}_{23} = \overline{Y}_{13} = 1p.u.$, as shown in the top right corner of Supplementary Fig. 4a. Node 1 represents a sub-station, from which electricity generation is injected and Nodes 2 and 3 are transformers connecting the consumers with load demands. The names of nodes e.g., 1, 2, and 3 are replaced by C, A, and B respectively. Actually, the sub-station node is regarded as a reference/slack bus and the other nodes are load buses. It should be noted that the unit, per unit ($p.u.$), is the expression of system quantities as fractions of a defined base unit quantity. The detailed definition is introduced in Supporting Information. **(a) Safe operation boundary (SOB) of a DG with two consumers located at nodes A and B.** If the power demand on the grid falls beyond the blue outline (SOB) then the DG collapses. **(b) SOB of the DG and consumers' power demand.** Scatter indicates DG operating points, where points within the boundary are safe operating points and those outside the boundary are unsafe operating points. An ellipse of 90% confidence intervals (CI) is used to indicate the joint distribution of consumer power demand. The ratio of the area (shaded region) of the green ellipse (joint distribution) outside the blue outline to the total area of the green ellipse indicates the probability of DG collapse. **(c) Impact of climate change on the SOB of DG and consumer demand.** Due to global climate change, the SOB shrinks, while the joint distribution of power demand shifts upwards (demand increases). This phenomenon of the SOB and ellipse moving away from each other due to climate change causes an increase in the risk of DG collapse. Source data are provided as a Source Data file.

frequent and severe. Literature has forecasted that climate change will induce more heating and cooling hours [11]. In the same cooling hours, the consumers have a higher probability of using more electricity in the future than nowadays [12]. According to supplementary equation (1), higher electricity demand results in the higher probability $\Phi[P|T_i, h] > 0$, therefore the blackout risk of the DG system increase. For instance, if climate change causes the temperature increase in the triangle DG system, the joint distribution of the demands on Nodes 2 and 3 moves toward the high-demand direction. Consequently, the probability of blackout increases.

We found that an increase in temperature from $25°C$ to $35°C$ only shifted the SOB inwards by 0.01 $p.u.$. We estimate the effect of the temperature increase and found that the effect is secondary. The risk increase caused by the transmission-capacity decline is much smaller than that caused by the demand growth during the temperature increase. Therefore, we ignore the effect of GCC on the transmission line capacity in the following discussion.

To study the effect of temperature on the DG's safe operation boundary (SOB), a triangular three-bus network is considered. The SOB is dependent on the conductance and susceptance of the transmission lines connecting the buses to each other in Supplementary Fig.2 of the main part, which are represented with variables $\overline{Y}_{12}$, $\overline{Y}_{23}$ and $\overline{Y}_{13}$. The lower the conductance of a transmission line, the less current flows through it and thus the lower amount of power flows through it. According to Temperature Coefficient Formula [13], the conductivity of a metal decreases with temperature. This means that the power flow in a transmission line decreases as the temperature increases.

Here, we are considering a normal temperature increase scenario ($25°C$ and $35°C$) on the transmission line. All the conductors are made up of Aluminum Conductor Composite Core (ACCC) conductors in this simulation. 1-mile length of conductor is considered, and all the 3 conductors have a uniform diameter of 28.1 mm. Therefore, the AC resistance of ACCC in $25°C$ is 0.089 ohms, and that in $35°C$ is 0.0925 ohms. In the simulation, the conductance Y reduces from 1 $p.u.$ to 0.962 $p.u.$.

As shown in Supplementary Fig. 5, the boundaries in $25°C$ and $35°C$ have not changed significantly. The $35°C$-boundary is only 0.01 $p.u.$ inward from $25°C$-boundary. Therefore, the effect of temperature on transmission line capacity is limited. We also observed that the local distribution SCADA centers are more concerned with the changes in the load demand instead of the line parameter themselves. Ref.[14] also mentioned that the change in line parameters is only a concern when there are wildfires (nature physics event) but does not mention the de-rating of lines due to climate change which is a much lower impact in terms of temperature change.

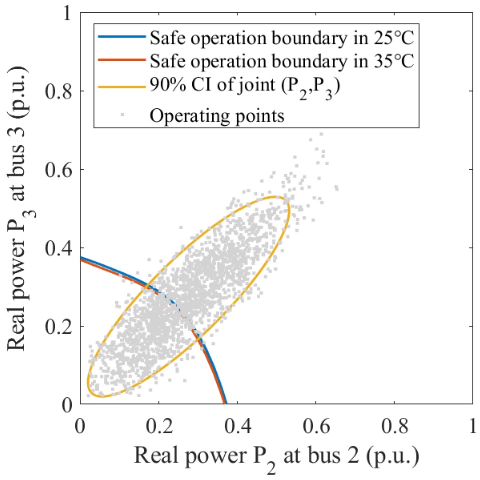

Supplementary Fig. 5 **The safe operating boundaries in** 3**-bus mesh network 25°C and 35°C.** The yellow circle represents the 90% confidence intervals (CI) of Joint (P_2, P_3), indicating the 90% confidence interval for real power at buses 2 and 3. The blue line marks the Safe Operation Boundary at 25°C, while the red line shows the Safe Operation Boundary at 35°C, defining safe limits at different temperatures. The black dots are operating points, showing specific system states. Source data are provided as a Source Data file.

Supplementary Note 6. Exploring risk uncertainty and method validation

The sensitivity analysis we conducted involves varying multiple parameters to understand their impact on the overall risk estimation. Specifically, we looked at different climate models (20 CMIP5 climate models) and scenarios (two Representative Concentration Pathway (RCP) scenarios) to capture the probabilistic nature of climate change impacts. We also considered variations in consumer distribution over the DG network to account for the uncertainty in how electricity is distributed and consumed. Supplementary Fig.6 presents the maximum and minimum risks in each year under RCP 4.5 and RCP 8.5.

It's important to note that the error bars in Supplementary Fig.6 represent the range of uncertainty around our risk estimates. This uncertainty arises from several sources, including the inherent uncertainty in climate modeling and the variability in energy consumption patterns.

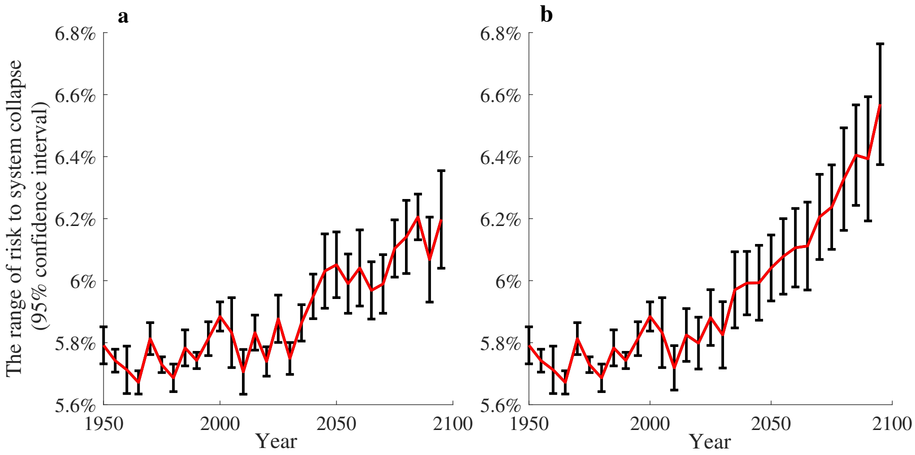

Supplementary Fig. 6 The figure shows the range of risk estimates for each year under Representative Concentration Pathway (RCP) 4.5 (a) and RCP 8.5 (b). The error bars represent the uncertainty arising from variations in 20 Coupled Model Intercomparison Project Phase 5 (CMIP5) models and scenarios, as well as consumer distribution over the distributed grid. Source data are provided as a Source Data file.

To rigorously validate the efficacy of our proposed method, we conducted a comprehensive examination of operating points and their associated risks within historical data spanning several years, as shown in Supplementary Fig. 7. Our investigation revealed a compelling trend: our method consistently reveals significantly elevated outage risks under circumstances of heavy load. This heightened risk corresponds to a heightened probability of voltage violations [**Weng**], a crucial concern in power systems operation.

This observed connection can be attributed to the robust foundation upon which our method is built. By drawing insights from grid topology, system parameters, and the power flow equation, our approach accounts for a diverse range of critical factors. In particular, it accurately captures the intricate interplay between load conditions and system vulnerabilities,

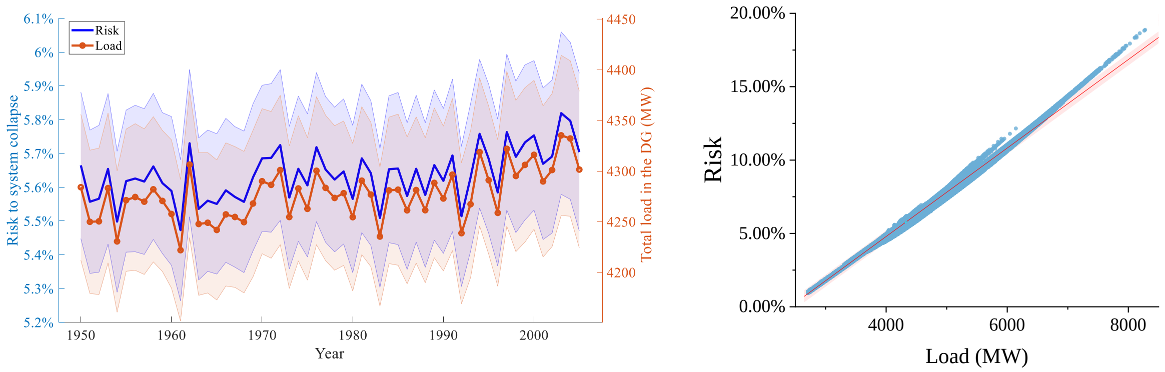

(a) Annual trend illustrating blackout risk and total load (b) The association between load and risk

Supplementary Fig. 7 The correlation between total load and predicted risk based on historical data from 1950 to 2005. (a) Annual trend illustrating blackout risk and total load. The data for each year includes operational data for typical days of 24 hours across 12 months. The blue and orange solid lines respectively represent the mean annual operational risk and the total distribution load, with shaded areas indicating the standard deviation of the 288 operational data points for each year. (b) The association between load and risk. Blue points represent operational points reflecting total load and predicted risk. The shaded red line depicts the linear regression function. This significant linear relationship effectively demonstrates the validity of our proposed method. It highlights great outage risks when the loads are large.

enabling us to pinpoint scenarios where the grid is most susceptible to failures.

Supplementary Note 7. Data Introduction

We provide information relevant to the quality and nature of the data that is used to assess the system risk in this article. The information required to compute the risk of the DG are 1) the topology of the DG along with its distribution lines' admittance values, 2) hourly power consumption data of the consumers at every node in the DG, and 3) hourly temperature data corresponding to the geo-location where the power consumption data is retrieved. 4) load temperature response function for 33 countries in Europe and 47 states in U.S.

Distribution grid data: The DG information used in this paper originates from a distribution system near the coast of California. This information includes the topology data such as nodes and edges. It also contains the admittance values of the edges in the graph-like structure of the DG. The available DG data with six different primary distribution feeders originating from a single substation is shown in Supplementary Information. The total number of nodes on each feeder according to the lexicographic order of their feeder names are $293, 953, 1104, 1289, 440,$, and 473 respectively. In this work, we present the results of analyzing the risk of Feeder 1 topology. It is also assumed that the Feeder 1 is balanced since the analysis is considered to be on the primary side of the distribution system. The standard distribution model is selected as IEEE-33 buses distribution system [15].

Load consumption data: For micro analysis, we have load data from Feeder 1 with 83 unique load profiles at upstream of the transformer of a real-world distribution system in California. The resolution of the load consumption data is hourly, using a total of four years of data from 2015 to 2019. All the hourly load data points for each consumer are tagged with a geolocation and a time stamp.

For macro analysis, the load data are disclosed by European Network of Transmission Systems Operators for Electricity [16] for different countries from 2006 to 2023 for 8760 hours. For the U.S., we extracted the corresponding minimum and maximum loads for each company from the original databases, the Federal Energy Regulatory Commission Form 714 (FERC-714)[17] and Energy Information Administration Form 861 (EIA-861)[18]. The specific data processing is in Supplementary Information.

Historical temperature data: Using the geolocation and timestamp of the hourly load data points of each consumer from the real-world consumer database, the historical hourly temperature data experienced by each consumer at the same time from 2015 to 2019 are collected from the nearest weather stations. This is collected using a weather API developed by the NOAA [19]. Using the geolocation and timestamp tagged load consumption and temperature data of different consumers from real-world consumer databases and real-world weather stations of NOAA, respectively. The conditional distributions of each consumer's demand given the temperature (temperature-load response curves) are learned for different consumers. The learning process of conditional distribution is further discussed in Supplementary Information. For the assessment of Europe and the United States, historical temperature curves are not required since they are used to fit the load-temperature response curves. Instead, we directly obtained these curves from the literature.

Load temperature response function: For Europe, we utilized response functions of country-level-aggregated peak load versus daily average temperature, as disclosed by Leonie Wenz [20]. The response function not only combines the responses covered by the data sample to derive the relationship between electricity consumption/peak load and temperature but also calibrates the response function of load and temperature to predict electricity consumption/peak load beyond the current

temperature range experienced by each country. Meanwhile, for the United States, we employed the response functions of electricity peak load versus daily average temperature, as disclosed by Maximilian Auffhammer [21]. The response function is capable of extrapolating beyond the observed temperature distribution to accurately estimate the changes resulting from extreme temperature increases due to climate change by focusing on the ”tails” of the temperature distribution.

Supplementary Method

Supplementary Method 1. The climate change models

The 20 models obtained correspond to the geolocation of real-world DG (distributed generation) locations in California, which were selected based on their similarity to the DG locations in the simulated dataset. These locations were then used to evaluate the performance of the 20 CMIP5 models. The specific 20 CMIP5 models used in the evaluation are shown in Supplementary Tab. 1.

Supplementary Tab. 1 20 Coupled Model Intercomparison Project Phase 5 (CMIP5) Models

NorESM1-M	bcc-csm1-1-m	bcc-csm1-1	BNU-ESM	CanESM2
CCSM4	CNRM-CM5	CSIRO-Mk3-6-0	GFDL-ESM2G	GFDL-ESM2M
HadGEM2-CC365	HadGEM2-ES365	inmcm4	IPSL-CM5A-LR	IPSL-CM5A-MR
IPSL-CM5B-LR	MIROC5	MIROC-ESM	MIROC-ESM-CHEM	MRI-CGCM3

According to climate change scenarios RCP 4.5 and RCP 4.5, which represent different levels of greenhouse gas emissions, the seasonal average temperature forecast for California has been projected, as shown in Supplementary Fig. 8. The historical period ending in 2005 and the forecast starting from 2006 have been considered. The average temperature forecast for the historical scenario is represented by the black line. The blue line represents the temperature forecast for the RCP 4.5 scenario, and the red line represents the temperature forecast for the RCP 4.5 scenario.

The forecasts have been generated by analyzing the data from 20 CMIP5 models and their standard deviation has been represented by the corresponding shaded envelopes. The results suggest that under the RCP 4.5 scenario, there could be a significant increase in temperature in California in the coming years, while under the RCP 4.5 scenario, the increase could be relatively lower.

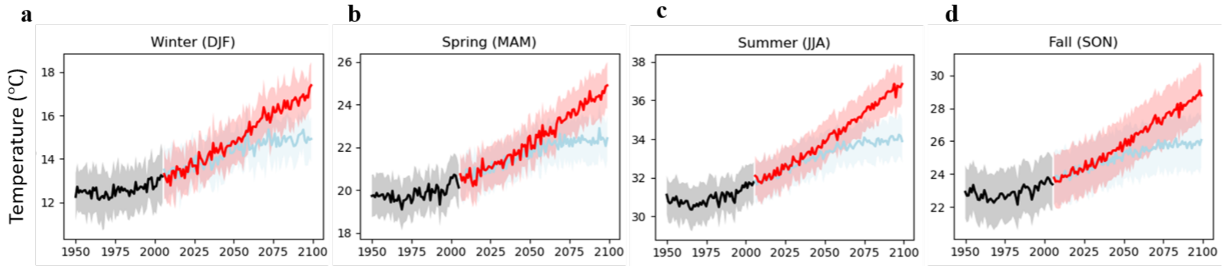

Supplementary Fig. 8 **Temperature forecast due to greenhouse gas emissions considering Representative Concentration Pathway (RCP) 4.5 and RCP 4.5 climate change scenarios.** Each panel (a-d) represents a seasonal average: (a) winter, (b) spring, (c) summer, and (d) autumn. Seasonal average temperature forecast of California for historical (black), RCP 4.5 (blue), and RCP 4.5 (red) scenarios considering climate change [26,27]. The historical period ends in 2005 and the forecast begins in 2006. The solid line indicates the average of 20 Coupled Model Intercomparison Project Phase 5 (CMIP5) models and their standard deviation is indicated by the corresponding shaded envelopes.

Supplementary Method 2. Generation of Load Curves in Europe and the United States

The generation of future load curves relies on the integration of two sets of data: load temperature response functions (U-shaped curves) and future temperature prediction data. In this paper, we present the acquisition and processing methods for both datasets to obtain load forecasts for the United States and Europe under RCP 4.5 and RCP 8.5 scenarios.

Demand Temperature Response Function

For Europe, we utilized regression curves of country-level-aggregated peak load versus daily average temperature, as disclosed by Leonie Wenz [20]. Meanwhile, for the United States, we employed the response functions of electricity peak load versus daily average temperature, as disclosed by Maximilian Auffhammer [21]. Given that counties within each state in

the U.S. are served by different electric utilities, we selected representative U-shaped curves of typical power companies within each state as the load temperature response functions. The correspondence between the power companies and states is presented in Supplementary Tab. 2. We retrieved the corresponding data using the *getdata* tool and performed curve fitting to obtain the U-shaped curves for each state and each country.

Supplementary Tab. 2 The correspondence between states and electric utilities.

Respondent ID	EIA Code	Utility Name	State
102	195	Alabama Power Co	Alabama
105	20856	Wisconsin Power & Light Co	Wisconsin
106	9392	Alliant Energy-West	Iowa
107	9208	Ameren (Illinois Power Co. Control Area)	Illinois
108	19436	Union Electric Co - (MO)	Missouri
116	803	Arizona Public Service Co	Arizona
142	3265	Cleco Power LLC	Louisiana
143	3989	City of Colorado Springs - (CO)	Colorado
149	4716	Dairyland Power Cooperative	Maryland
150	4922	Dayton Power & Light Co	Ohio
269	5109	DTE Electric Company	Michigan
157	5416	Duke Energy Carolinas, LLC	North Carolina
171	6452	Florida Power & Light Co	Florida
174	7490	Grand River Dam Authority	Oklahoma
119	9191	Idaho Power Co	Idaho
190	10015	Westar Energy (KPL)	Kansas
197	11249	Louisville Gas & Electric Co	Kentucky
200	12293	City of Memphis - (TN)	Tennessee
203	12431	MidAmerican Energy Company	South Dakota
210	13407	Nevada Power Co	Nevada
215	13756	Northern Indiana Pub Serv Co	Indiana
216	13781	Northern States Power Co - Minnesota	Minnesota
217	12825	NorthWestern Energy LLC - (MT)	Montana
220	14063	Oklahoma Gas & Electric Co	Arkansas
223	14127	Omaha Public Power District	Nebraska
227	14328	Pacific Gas & Electric Co	California
231	15143	Platte River Power Authority	Wyoming
232	15248	Portland General Electric Co	Oregon
139	15500	Puget Sound Energy Inc	Washington
251	17543	South Carolina Public Service Authority	South Carolina
259	17718	Southwestern Public Service Co	New Mexico
285	12658	Minnkota Power Cooperative, Inc.	North Dakota
162	7140	Georgia Power Co	Georgia
297	12686	Mississippi Power Co	Mississippi
/	/	CT (Connecticut)	Connecticut
/	/	ME (Maine)	Maine
/	/	NH (New Hampshire)	New Hampshire
/	/	RI (Rhode Island)	Rhode Island
/	/	VT (Vermont)	Vermont
/	/	WCMASS	Massachusetts
/	/	NYISO	New York
/	/	ERCOT	Texas
/	/	ATSI	Delaware
/	/	PL	New Jersey
/	/	GPU	Pennsylvania
/	/	RECO	Virginia
/	/	AEP	West Virginia

The source of European data in the paper [20] is described as follows: The dataset for Europe includes a time series of population-weighted daily maximumand daily average temperature for each of the 33 European countries for the years 2006-2012 (observational data) and 2013-2099 (projected values under different scenarios of climate-change mitigation). Population projections used for within-country temperature weighting correspond to the "middle-of-the-road" Shared Socio-economic Pathway (SSP-2 [22]). All temperature and population data come from the climate dataset of the Intersectoral

Impact Model Intercomparison Project (SI-MIP [23]) covering bias-corrected [24] data on a horizontagrid of 0.5°resolution. The European Network of Transmission Systems Operators for Electricity [16] provides dataon electricity load consumption (i.e., hourly load values per country) for the period 2006-2012. As a result of this comprehensive data compilation, the generated U-shaped curve is readily employable. This curve seamlessly integrates demographic insights, historical data, and future load forecasts.

In the dataset, U-shaped load curves for all 33 European countries are complete with the corresponding axis, allowing direct usage. However, in the case of the United States, only two complete U-shaped load curves with their corresponding axis, namely Texas and PJM, were disclosed. For the remaining 166 different utilities, only the curve was presented without the axis. Thus, we adopted a reasonable approach to reconstruct the original load-temperature response curves.

To achieve this, we used the original x-axis from the published figures as the x-axis for all images and aligned them with the black dashed lines in the original graphs. Following the description provided by Maximilian Auffhammer, we identified the bottom of each U-shaped curve as corresponding to the minimum load between 2006 and 2014. Therefore, we extracted the corresponding minimum and maximum loads for each company from the original databases, the Federal Energy Regulatory Commission Form 714 (FERC-714)[17] and Energy Information Administration Form 861 (EIA-861)[18]. The bottom of each U-shaped curve was set to match the minimum load value, and the top of the curve was set at 1.15 times the maximum load value. The final restored U-shaped curve is shown in Supplementary Fig. 9. It is important to note that in the final analysis, we scaled the loads down to IEEE-33 level loads for comparative purposes. As a result, the shape of the U-shaped curve itself is of utmost importance, and any deviation in the vertical axis will not have a substantive impact on the results.

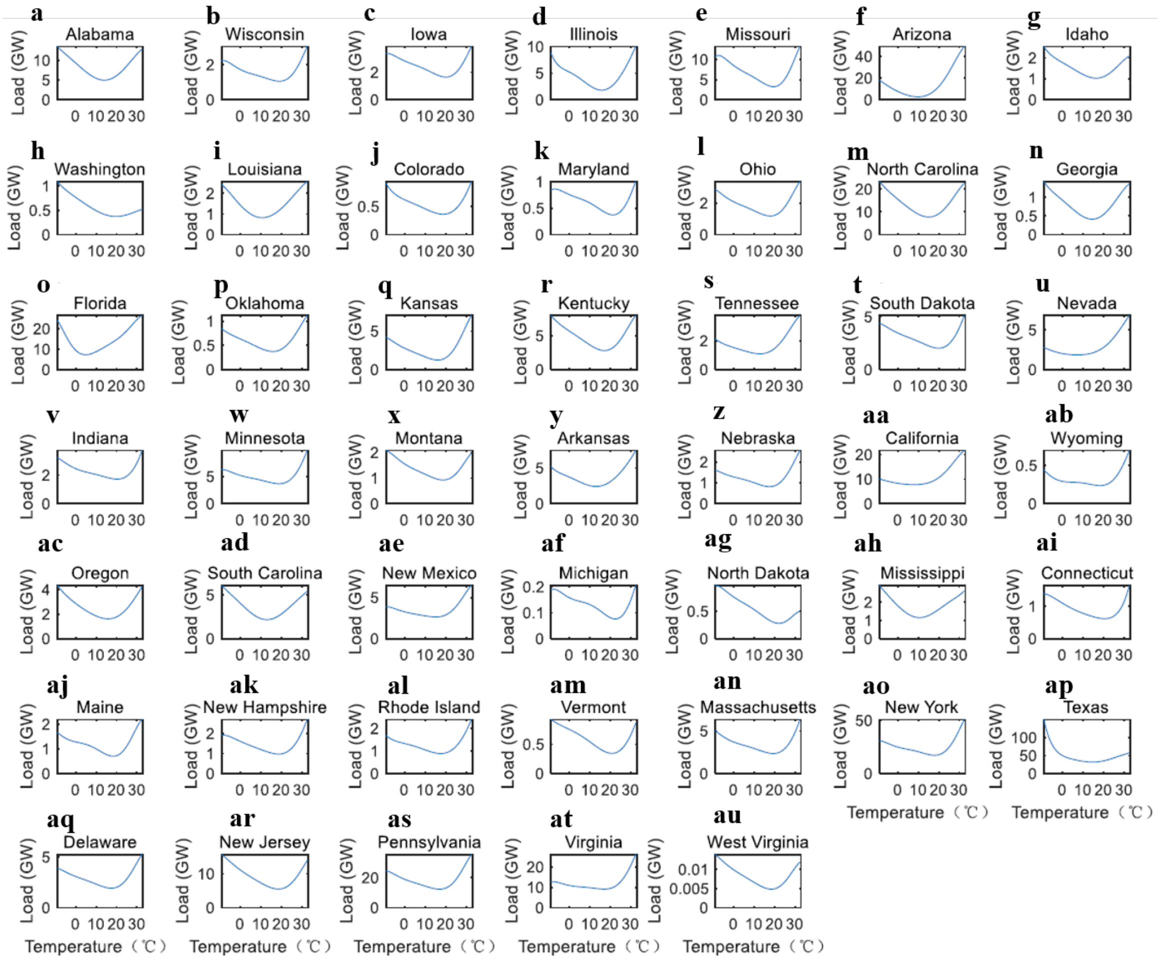

Supplementary Fig. 9 **Reconstructed U-shaped load-temperature response curves for the United States.** Each panel (labeled a-au) represents a U.S. state, with the state name labeled above the panel. The curves in each panel represent the relationship between temperature and electricity load for 47 different utilities, with the x-axis derived from the original published figures and aligned using black dashed lines. The bottom of each U-shaped curve corresponds to the minimum load between 2006 and 2014, while the top is set at 1.15 times the maximum load value, as extracted from the Federal Energy Regulatory Commission Form 714 (FERC-714) and Energy Information Administration Form 861 (EIA-861). The curves were scaled down to IEEE-33 level loads for comparative analysis, ensuring that the shape of the curve, rather than the vertical axis, is the primary focus. Source data are provided as a Source Data file.

Future temperature prediction data

The temperature data for the United States is sourced from the National Climate Change Viewer (NCCV) [1], an official temperature forecast dataset provided by the United States Geological Survey (USGS). The USGS NCCV dataset includes the historical and future climate projections from 20 of the downscaled models for two of the RCP emission scenarios [1]. The dataset includes the temperature trajectory of all counties in the US. Two climate-change scenarios proposed by IPCC are considered, which are the RCP 4.5 and RCP 8.5 scenarios. The specific 20 CMIP5 models are shown in Supplementary Tab. 1. The mean values of downscaled 20 CMIP5 models are shown in Supplementary Fig. 8.

For the European temperature data, we accessed the Climate Change Knowledge Portal (CCKP)[25], which is a comprehensive dataset established by the World Bank to provide global, regional, and national data related to climate change and development. The CCKP includes average temperatures for European countries under different RCP levels from 2005 to 2100, as well as temperature prediction curves for 12 months in four time periods: 2020-2039, 2040-2059, 2060-2079, and 2080-2099. To derive the average monthly temperatures for each year, we use the annual average temperature and the temperature prediction curves for the respective time periods. To calculate the temperature for each month (x_i) in the year 2021, we use supplementary equation (2):

$$x_i = \frac{z}{\text{mean}(y)} \cdot y_i \tag{2}$$

where, x_i represents the temperature for the ith month in 2021. z is the average temperature data for the year 2021. y_i is the temperature value for the ith month in the temperature prediction curve for the respective time period (2020-2039, 2040-2059, 2060-2079, or 2080-2099). $\text{mean}(y)$ is the average value of the temperature data for all 12 months in the temperature prediction curve.

Next, to generate the load data, we use the selected U-shaped curves where the x-axis represents the temperature and the y-axis corresponds to the peak load for each 24-hour period. We input the average temperature for each day of the year into the load-temperature response curve to obtain the maximum load for each day. Then, based on the load data disclosed on the website ”Power Statistics” [16] for different countries from 2006 to 2023 for 8760 hours, we average the load data for all 24-hour periods to create the standard 24-hour load curve. By combining the peak load for each day with the normalized load curve for that region, we obtain the 24-hour load data for each day. To calculate the load data for each 24-hour period ($L2_i$), we use supplementary equation (3):

$$L2_i = \frac{P_L}{\max(L1)} \cdot L1_i \tag{3}$$

where, $L2_i$ represents the load prediction data for the ith hour of that day. P_L is the peak load for that day. $L1_i$ is the load data for the ith hour in the standard 24-hour load curve. $\max(L1)$ is the maximum load in the standard 24-hour load curve. These equations allow us to predict the temperature for each month in 2021 and generate the load data for each 24-hour period, facilitating our analysis of future energy demands in Europe and the United States.

Supplementary References

1. Alder, J. R. and Hostetler, S. W. (n.d.). *USGS National Climate Change Viewer. US Geological Survey*. https://doi.org/10.5066/F7W9575T. (2013).
2. National conference of state legislatures (n.d.). *Modernizing the Electric Grid: State Role and Policy Options*. https://www.ncsl.org/energy/modernizing-the-electric-grid#:~:text=Sixty%20percent%20of%20U.S.%20distribution,grid%20just%20to%20maintain%20reliability. (2021).
3. Tokarska, K. B., Gillett, N. P., Weaver, A. J., Arora, V. K., and Eby, M., The climate response to five trillion tonnes of carbon. *Nat. Clim. Change* **6**, 851–855 (2016).
4. Warren, R., VanDerWal, J., Price, J., Welbergen, J. A., Atkinson, I., Ramirez-Villegas, J., Osborn, T. J., Jarvis, A., Shoo, L. P., Williams, S. E., et al., Quantifying the benefit of early climate change mitigation in avoiding biodiversity loss. *Nat. Clim. Change* **3**, 678–682 (2013).
5. Wadhwa, C., *Electrical power systems*. New Age International, (2006).
6. “Fundamentals of Electric Power” (n.d.). In: *Renewable and Efficient Electric Power Systems*. Chap. 2, 51–105. DOI: https://doi.org/10.1002/0471668826.ch2.
7. Ochoa, L. F., Dent, C. J., and Harrison, G. P., Distribution Network Capacity Assessment: Variable DG and Active Networks. *IEEE Trans. Power Syst.* **25**, 87–95 (2010).
8. Vallée François, O. S. and Abdeljebbar, C., Optimal Allocation of Combined Renewable Distributed Generation and Capacitor Units for Interconnection Cost Reduction. *J. Electr. Comput. Eng.*, 5101387 (2020).
9. Weng, Y., Rajagopal, R., and Zhang, B., A geometric analysis of power system loadability regions. *IEEE Transactions on Smart Grid* **11**, 3580–3592 (2019).
10. Li, Y., Pizer, W. A., and Wu, L., Climate change and residential electricity consumption in the Yangtze River Delta, China. *Proc. Natl. Acad. Sci.* **116**, 472–477 (2019).

11. Intergovernmental Panel on Climate Change (IPCC), *Climate Change 2014: Impacts, Adaptation, and Vulnerability. Part B: Regional Aspects. Contribution of Working Group II to the Fifth Assessment Report of the Intergovernmental Panel on Climate Change* (2014).
12. Hossain, M. S., Pota, H., Mahmud, M. A., and Saha, T., Impact of extreme weather conditions on energy consumption in residential households in Australia. *Energy Build.* **174**, 516–527 (2018).
13. Ashby, M. F. and Jones, D. R. H., *Engineering Materials 1: An Introduction to Properties, Applications and Design*. Elsevier, (2013).
14. Dumas, M., Kc, B., and Cunliff, C. I., *Extreme Weather and Climate Vulnerabilities of the Electric Grid: A Summary of Environmental Sensitivity Quantification Methods* (2019).
15. Dolatabadi, S. H., Ghorbanian, M., Siano, P., and Hatziargyriou, N. D., An enhanced IEEE 33 bus benchmark test system for distribution system studies. *IEEE Trans. Power Syst.* **36**, 2565–2572 (2020).
16. European Network of Transmission System Operators for Electricity (ENTSO-E), *ENTSO-E Power Statistics*, URL: https://www.entsoe.eu/data/power-stats/, (2023).
17. Federal Energy Regulatory Commission (FERC), *Form No. 714: Annual Electric Balancing Authority Area and Planning Area Report*, URL: https://www.ferc.gov/industries-data/electric/general-information/electric-industry-forms/form-no-714-annual-electric/overview, (2023).
18. U.S. Energy Information Administration (EIA), *EIA-861: Annual Electric Power Industry Report*, URL: https://www.eia.gov/electricity/data/eia861/, (2022).
19. National Oceanic and Atmospheric Administration, *Historical and Forecast API for Weather data*, URL: https://www.weather.gov/documentation/services-web-api, (2019).
20. Auffhammer, M., Baylis, P., and Hausman, C. H., Climate change is projected to have severe impacts on the frequency and intensity of peak electricity demand across the United States. *Proc. Natl. Acad. Sci.* **114**, 1886–1891 (2017).
21. Wenz, L., Levermann, A., and Auffhammer, M., North–south polarization of European electricity consumption under future warming. *Proc. Natl. Acad. Sci.* **114**, E7910–E7918 (2017).
22. Moss, R. H., Edmonds, J. A., Hibbard, K. A., Manning, M. R., Rose, S. K., Van Vuuren, D. P., Carter, T. R., Emori, S., Kainuma, M., Kram, T., et al., The next generation of scenarios for climate change research and assessment. *Nature* **463**, 747–756 (2010).
23. Frieler, K., Levermann, A., Elliott, J., Heinke, J., Arneth, A., Bierkens, M., Ciais, P., Clark, D., Deryng, D., Döll, P., et al., A framework for the cross-sectoral integration of multi-model impact projections: land use decisions under climate impacts uncertainties. *Earth Syst. Dyn.* **6**, 447–460 (2015).
24. GADM, *GADM - Global Administrative Areas*, URL: https://gadm.org/download_country.html, (2014).
25. World Bank, *Climate Change Knowledge Portal (CCKP)*, URL: https://climateknowledgeportal.worldbank.org/, (2021).
26. Riahi, K., Grübler, A., and Nakicenovic, N., Scenarios of long-term socio-economic and environmental development under climate stabilization. *Technological Forecasting and Social Change* (2007).
27. Clarke, L. E., *Scenarios of greenhouse gas emissions and atmospheric concentrations: report*. US Climate Change Science Program, (2007).
